# Wilfred Martin Barclay

**Published:** 1903-06

**Authors:** 


### Obituary Notices.

### WILFRED MARTIN BARCLAY, F.R.C.S. ENG.

It is with much regret that we have to record the death of Wilfred Martin Barclay, of Clifton. He died at Amberley, in Gloucestershire, of pulmonary tuberculosis, after a few months' illness. His death came rather as a surprise to most of his friends, who had not realised that his end was so near. Mr. Barclay was born in India on May 15th, 1863, and died on May 9th last, and was therefore at the time of his death only a few days short of his fortieth year. He was the youngest son of the late Deputy Surgeon-General George Barclay, of the Madras army; he was educated at Clifton College, laying there the foundation of those literary tastes which became more fully developed as years went on, and in the pursuit of which he took a keen interest and pleasure. As to his literary qualifications, Canon Ainger writes for the purposes of this notice: "He had a very marked liking and taste for good literature, especially for poetry and the drama, and a most just and proper contempt for much that passes for fine writing and profound philosophy in our day; in fact, he had a very fine and strong sense of humour, which is the best of safeguards against being taken in by the charlatans of the hour. He was in consequence one of the most charming of companions, and he leaves a blank in my Bristol life which no one can fill." Canon Ainger also adds: "During the thirteen years that I knew him he had suffered many grievous family bereavements, and lived through years of much loneliness and anxiety; and when at last he made the most congenial and happy of marriages, his friends hoped that a long future of domestic happiness lay before him, but *Deo aliter visum.*"

Mr. Barclay received his professional education at the Bristol University College and the Bristol General Hospital, and was successful in gaining several prizes during his student career. Soon after obtaining the membership of the College of Surgeons, he was elected Assistant House-Surgeon to the General Hospital, and in the year 1888 he was appointed Assistant Surgeon. At the time of this appointment the majority of the staff very strongly felt that only Fellows of the College should be eligible for honorary surgical posts, but, notwithstanding this, Mr. Barclay was elected by the Governors on the understanding that he should become a Fellow within twelve months, or resign the office. Mr. Barclay succeeded in passing both the primary and final examinations within nine months, an achievement which gained for him the esteem and goodwill of most of those who were not at first favourable to his candidature; he was elected Surgeon in 1893. His promotion was singularly rapid, as after little more than eight years, and at the early age of 38, he was Senior Surgeon to the Hospital. He was less fortunate with regard to the Medical School; for though he was for a time Demonstrator of Anatomy, he had always someone in front of him with a prior claim when a lectureship became vacant. Mr. Barclay was only an occasional contributor of papers or a speaker at our medical meetings, but he was much interested in this *Journal*, and for several years was a regular contributor to our reports on surgical progress. He was Honorary Surgeon to the Bristol Nurses' Institution, taking a keen interest in the efficient training and proper teaching of nurses, and giving for some time regular lectures to the nursing staff of the General Hospital.

Mr. Barclay was an excellent surgeon and a very good operator, unsparing in the care he gave to his patients and very careful as to detail; indeed, if he had a fault as an operator, it was over minuteness of detail with its consequent prolongation of the operation, but this was perhaps more due to modern methods than to the individual.

Mr. Barclay was most kind and cheery to those under his charge, somewhat reticent and retiring to his more superficial acquaintances, but he was a most pleasant and agreeable companion to those who knew him best: he seemed to prefer a small circle of congenial friends to the amenities of the wider social circle. He was a gentleman in the best sense—refined, high minded, cultivated, incapable of anything mean or petty, and he was intolerant of any suspicion of such things in others. He had formed for himself a high ideal of life, but was perhaps a shade over-sensitive to grievances, real or imaginary, which fall to the lot of most of us in the rough and tumble of the world.

Such was Wilfred Barclay as he appeared to the writer.

> "His life was gentle, and the elements
> So mix'd in him that Nature might stand up
> And say to all the world, 'This was a man!'"

